# Pituitary abscess, plexus-choroiditis and encephalitis by *Helcococcus ovis* in a calf

**DOI:** 10.1007/s11259-026-11171-x

**Published:** 2026-03-21

**Authors:** Jean Carlo Olivo Menegatt, Ellen Cristina de Oliveira, Cassiane Elisabete Lopes, Fernanda Felicetti Perosa, Eduarda Keil, Jeniffer Bomfim Carvalho, Mariana Bandeira Casagrande, David Driemeier

**Affiliations:** Faculdade de Veterinária, Setor de Patologia Veterinária, Universidade Federal do Rio Grande do Sul, Av. Bento Gonçalves 9090, Porto Alegre, 91540-000 RS Brazil

**Keywords:** Basilar empyema, Meningitis, Neurological signs, Nose ring

## Abstract

We report here the first confirmed case of a pituitary abscess in a calf caused by *Helcococcus ovis*, an emerging pyogenic bacterium in veterinary medicine. An 8-month-old calf showed progressive neurological signs following the use of a nose ring. Gross and histopathological examination revealed a large abscess in the pituitary gland accompanied by leptomeningitis, encephalitis, and plexus choroiditis. *Helcococcus ovis* was isolated from the pituitary abscess and identified by MALDI-TOF mass spectrometry with high confidence. Coccoid gram-positive bacteria were observed on gram stain of formalin-fixed tissues from leptomeningitis, plexus choroiditis and pituitary abscess lesions. Gross and histological features were indistinguishable from abscesses caused by other pyogenic organisms, highlighting the critical importance of bacteriological culture to diagnosis. This case expands the known pathogenic spectrum of *H. ovis* and suggests its inclusion in the differential diagnosis of suppurative central nervous system lesions in cattle.

## Background

Pituitary abscess, also referred to as pituitary abscess syndrome or basilar empyema, is a suppurative infection of the pituitary fossa characterized by the formation of purulent and/or abscedative lesions in this area. Although pathogenesis remains unclear, the condition is frequently associated with chronic inflammatory lesions of the nasal cavity, often secondary to trauma induced by use of nose rings (Fernandes et al. 2000; Constable et al. 2017; Konradt et al. 2017). Most cases have been reported in cattle, but sporadic cases have also been described in sheep and goats (Morin 2004; Helman 2010; Konradt et al. 2017; Miller and Porter 2022; Maroneze et al. 2025). *Trueperella pyogenes* have been demonstrated as the main isolated bacterium from this condition (Constable et al. 2017; Konradt et al. 2017; Miller and Porter 2022).

*Helcococcus ovis* is a gram-positive, coccoid, catalase-negative, facultatively anaerobic bacterium, first described in 1999 in the United Kingdom from two clinical cases in sheep, one involving lesion in the lungs, liver, and spleen, and the other associated with subclinical mastitis (Collins et al. 1999). Recently, *H. ovis* has gained increasing attention in veterinary medicine, particularly as a primary pathogen in cases of endocarditis in cattle (Post et al. 2003; Kutzer et al. 2008; Schulze et al. 2016; Kemper et al. 2020). Beyond endocarditis, this organism has also been implicated in a range of other pyogenic infections, including metritis and mastitis in dairy cows (Locatelli et al. 2013; Liu et al. 2022), arthritis and bursitis in calves (Jost and Sickinger 2021), and endocarditis and hepatitis in a Leghorn rooster (Crispo et al. 2017). Additionally, *H. ovis* has been isolated from respiratory tract lesions such as pleuritis, bronchopneumonia, and pulmonary abscesses in horse, sheep, and goats (Rothschild et al. 2004; Zhang et al. 2009; García et al. 2012).

In this report, we describe the first case of pituitary abscess and neurological clinical signs due to infection by *H. ovis* in a calf.

## Case presentation

An 8-month-old male calf from a farm in Viamão, Rio Grande do Sul, Brazil, presented with hyporexia, apathy, intermittent fever and progressive neurological signs, including motor incoordination and ataxia, lasting approximately eight weeks. No other animals in the herd (*n *= 20) exhibited clinical signs. It was reported that the clinical signs started three weeks after the owner put a nose ring in the calf. The animal received two intramuscular treatments with oxytetracycline (IM; 20 mg/kg, Terramicina^®^; Zoetis) administered in the neck region. Following treatment, transient clinical improvement was observed, with a reduction in apathy, resolution of fever, and attenuation of neurological signs. However, the clinical condition later worsened, progressing to lateral recumbency, paddling movements, and opisthotonus. Due to the poor prognosis, the calf was euthanized, and a field necropsy was promptly conducted by the Setor de Patologia Veterinária of the Universidade Federal do Rio Grande do Sul (SPV-UFRGS).

**Fig. 1:**
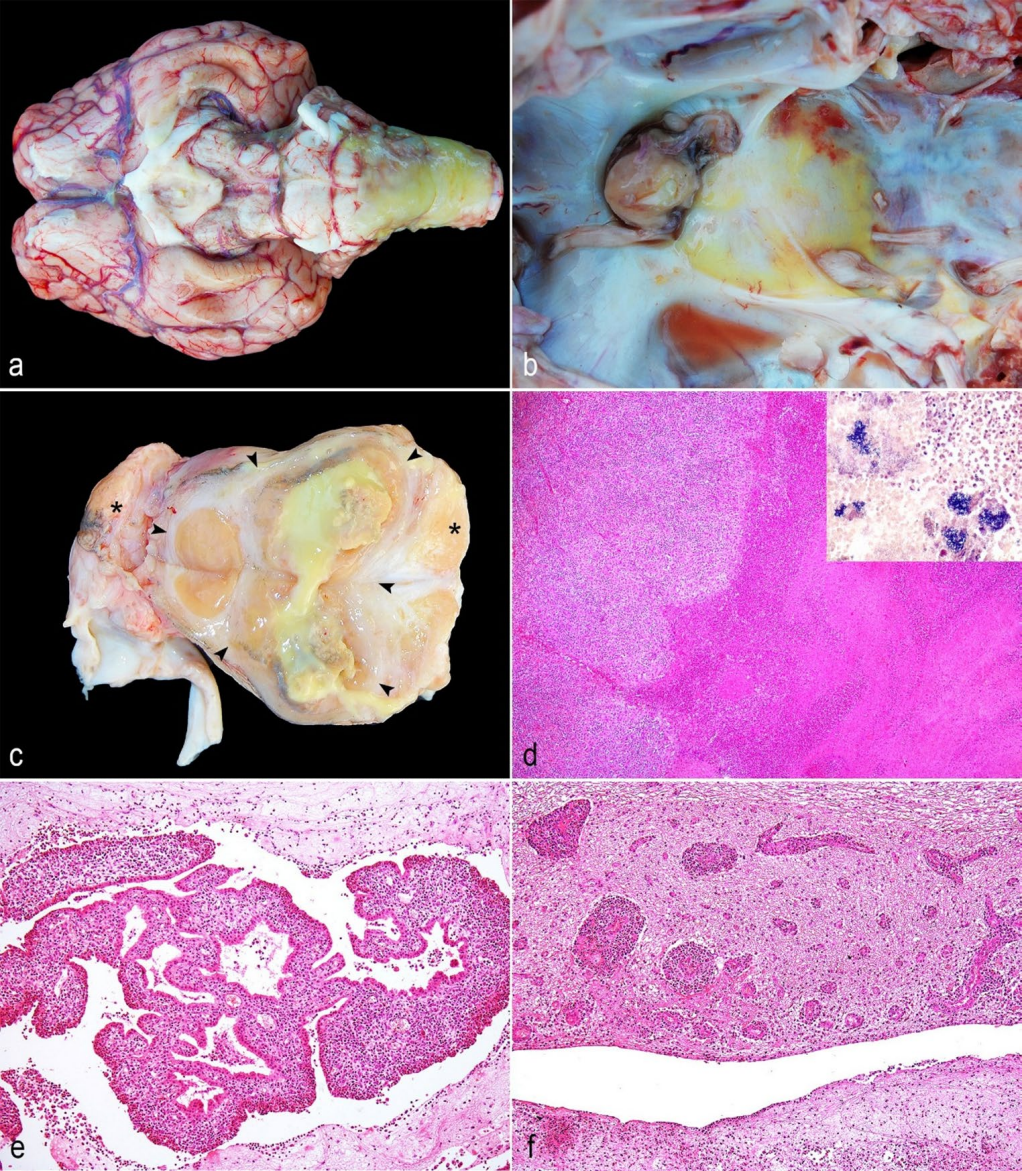
Gross and histopathological findings of pituitary abscess, plexus-choroiditis and encephalitis by *Helcococcus ovis* in a calf. **a** Basal region of the brain and cervical segment of the spinal cord presenting marked deposition of fibrin; **b** Marked enlargement and yellow discoloration of the pituitary fossa region due to a large abscess within the pituitary gland; **c** Cut surface of the pituitary gland showing abundant yellow purulent exudate within the pituitary parenchyma (abscess). Arrowheads delineate the abscess within the pituitary gland, and asterisks indicate the trigeminal ganglia; **d** Histological section of the pituitary gland showing a focally extensive area of central necrosis with cellular debris, fibrin, and degenerate neutrophils (liquefactive necrosis). HE. Obj. 4x. Inset: gram-positive bacteria aggregate within the pituitary abscess. Gram-stain. Obj. 40x; **e** Lateral ventricles and choroid plexus with marked inflammatory infiltrate of neutrophils and fibrin deposition. HE. Obj. 20x; **f** Neuropil and perivascular spaces in the periventricular region exhibiting multifocal mixed inflammatory infiltrates composed of neutrophils, lymphocytes, and macrophages. HE. Obj. 20x

At necropsy, the basal region of the brain (comprising diencephalon, mesencephalon, and rhombencephalon) and cervical segment of the spinal cord presented abundant fibrin deposition (Fig. 1a). The pituitary fossa was markedly enlarged and yellow due to a large pituitary abscess measuring 3 × 3 × 2.5 cm (Fig. 1b). On cut surface, the pituitary contained viscous yellow exudate surrounded by white fibrous tissue (abscedative lesion), which also involved the left side of the *rete mirabile* and the trigeminal ganglion (Fig. 1c). No relevant pathological changes were observed in the remaining tissues as well as in the basisphenoid bone, oral and nasal cavities. Samples of *rete mirabile*, pituitary gland, trigeminal ganglion, brain, spinal cord, lung, trachea, heart, esophagus, lymph nodes, liver, spleen, kidneys, small and large intestines, urinary bladder, gall bladder, skeletal muscle, nasal conchae, and skin were collected, fixed in 10% neutral-buffered formalin, and processed routinely for histologic examination. A 1 mL sample of purulent exudate from a basilar empyema involving the pituitary gland was collected and submitted for bacteriological examination.

Histologically, the *rete mirabile*, pituitary gland, and trigeminal ganglion, were markedly distended by focally extent area of liquefactive necrosis with infiltrates of degenerate neutrophils and surrounded by proliferation of fibrous connective tissue (abscedative lesion) (Fig. 1d). The leptomeninges of the basal region of the brain was thickened and presented large amounts of fibrin associated to numerous infiltrates of neutrophils, macrophages and lymphocytes that extended to the cervical segment of the spinal cord. The lateral ventricles and choroid plexus exhibited marked inflammatory infiltrate of neutrophils and fibrin deposition (Fig. 1e). Additionally, the neuropil and perivascular space of the periventricular region showed multifocal areas of mixed infiltrate inflammatory of neutrophils, lymphocytes, and macrophages (Fig. 1f). Within the pituitary abscess, leptomeningitis and choroid plexitis lesions, a mild to moderate number of coccoid bacteria were observed, which stained gram-positive with the Gram stain. No significant microscopic lesions were observed in other tissues.

After 48 h of incubation, non-hemolytic, gray, and sand-like pure colonies were observed on ovine 5% blood agar. No bacteria growth on MacConkey agar. The bacteria isolate was gram-positive. The final bacteria identification (*Helcococcus ovis*) was assessed by MALDI-TOF (Matrix-Assisted Laser Desorption Ionization Time-of-Flight) technique with a 2.06 score value, indicating high-confidence species-level identification.

### Discussion and conclusion

In a retrospective study conducted in Rio Grande do Sul, Brazil, basilar empyema accounted for 9.3% of suppurative central nervous system infections (CNS) in ruminants, with *Trueperella pyogenes* identified as the sole isolate in all cases (Konradt et al. 2017). This bacterium is considered the primary etiologic agent of basilar empyema in cattle, sheep, and goats and is frequently associated with suppurative or abscess-forming lesions in various tissues.

Grossly, abscesses caused by *Helcococcus ovis* are indistinguishable from those caused by other pyogenic bacteria, including *T. pyogenes*, *Corynebacterium* spp., *Staphylococcus* spp., and *Streptococcus* spp. Identification of *H. ovis* can be challenging due to its slow growth rate and morphological similarity to other gram-positive cocci. Conventional biochemical tests often yield inconclusive results, making molecular techniques such as 16 S rRNA gene sequencing or MALDI-TOF(Matrix-Assisted Laser Desorption Ionization Time-of-Flight) crucial for definitive identification and accurate differentiation of these bacteria (García et al. 2012; Mao et al. 2018).

Although the predisposing factors for infections caused by *Helcococcus* spp. are not yet fully elucidated, these bacteria are considered as commensals of keratinized epithelium in humans. In animals, previous studies have suggested that skin wounds may serve as a source of infection, with subsequent bacterial translocation and bacteremia (Rothschild et al. 2004; Crispo et al. 2017). In the present case, although the route of infection cannot be definitively established, the previous use of a nose ring is considered the most plausible source, as the majority of the studies of pituitary abscess have shown (Konradt et al. 2017; Miller and Porter 2022; Maroneze et al. 2025). No gross or microscopic lesions were observed in the nose and nasal cavities associated with nose ring use. However, the animal had a chronic clinical history and had received treatment, which may have led to complete resolution of an initial predisposing lesion, thereby obscuring evidence of the original portal of entry.

The reported mortality rate of pituitary abscess is low (usually < 2%), but the lethality is high, particularly without clinical treatment (Fernandes et al. 2000; Morin et al. 2004; Konradt et al. 2017; Miller and Porter 2022; Maroneze et al. 2025). Most cases are sporadic, although outbreaks have been described on farms where nose rings were used in calves (Fernandes et al. 2000; Morin et al. 2004). Following colonization of the pituitary region, the ensuing suppurative process may extend to the leptomeninges and adjacent brain parenchyma, resulting in leptomeningitis and encephalitis (Morin et al. 2004; Konradt et al. 2017; Miller and Porter 2022). Notably, inflammation of the choroid plexus (plexus choroiditis) was also observed, likely reflecting the severity and extent of the infection. In addition, the pronounced neurological signs observed clinically corresponded to the extensive brain lesions warranting the exclusion of differential diagnoses such as rabies, listeriosis, cerebral abscesses, among others. In this case, the combined use of gross pathology, histopathology, and bacteriological culture was essential for confirming the diagnosis.

The animal initially showed clinical improvement after tetracycline treatment. However, its clinical condition subsequently declined. Treatment failure in this case may be attributed to the extensive brain and pituitary lesions observed and poor penetration of the antibiotic into abscessed tissue, which can limit therapeutic efficacy. Furthermore, although antimicrobial susceptibility testing was not performed, previous studies have reported tetracycline resistance in *H. ovis* isolates from cattle, which may also explain the lack of a sustained therapeutic response (Bilk et al. 2011; Cunha et al. 2024).

Although *H. ovis* has increasingly been reported in various animal species, most studies have described its involvement in suppurative processes of the cardiorespiratory system (Post et al. 2003; Rothschild et al. 2004; Kutzer et al. 2008; Zhang et al. 2009; García et al. 2012; Schulze et al. 2016; Kemper et al. 2020). To our knowledge, this is the first case to describe *H. ovis* infection involving the pituitary region and the CNS producing neurological signs in animals. Additionally, the clinical presentation, gross pathology, and histological features were indistinguishable from those associated with more commonly implicated pyogenic bacteria.

This case underscores the critical importance of bacteriological culture and identification in establishing an accurate diagnosis. Furthermore, *H. ovis* should be considered in the differential diagnosis of pyogenic lesions, particularly in cattle, including those affecting the central nervous system.

**Statement of Animal Ethics** We authors of the article entitled “Pituitary abscess, plexus-choroiditis and encephalitis by *Helcococcus ovis* in a calf” declared, for all due purposes, the project was approved by the Research Committee (COMPESQ) of the Universidade Federal do Rio Grande do Sul under number 37,662. Thus, the authors assume full responsibility for the presented data and are available for possible questions, should they be required by the competent authorities.

## Data Availability

The data presented in this study are available on request from the corresponding author.
